# Parasite-Specific IL-17-Type Cytokine Responses and Soluble IL-17 Receptor Levels in Alveolar Echinococcosis Patients

**DOI:** 10.1155/2012/735342

**Published:** 2012-08-30

**Authors:** Christian J. Lechner, Beate Grüner, Xiangsheng Huang, Wolfgang H. Hoffmann, Peter Kern, Peter T. Soboslay

**Affiliations:** ^1^Institute for Tropical Medicine, University of Tübingen Clinics, 72074 Tübingen, Germany; ^2^Division of Infectious Diseases and Clinical Immunology, Comprehensive Infectious Disease Center, University of Ulm Hospitals, 89081 Ulm, Germany

## Abstract

Alveolar Echinococcosis (AE) caused by the cestode *Echinococcus multilocularis*, is a severe helminth infection of man, where unrestricted parasite growth will ultimately result in organ failure and fatality. The tissue-infiltrative growth of the larval metacestode and the limited efficacy of available drugs complicate successful intervention in AE; patients often need life-long medication, and if possible, surgical resection of affected tissues and organs. Resistance to AE has been reported, but the determinants which confer protection are not known. ln this study, we analyzed in patients at distinct stages of Alveolar Echirococcosis, that is cured, stable and progressive AE, as well as in infection-free controls, the cellular production and plasma levels of pro-inflammatory cytokines lL-17A, lL-17B, lL-17F and their soluble receptors lL-17RA (slL-17RA) and IL-17RB (sIL-17RB). Significantly elevated levels of IL-17B and slL-17RB were observed, whilst lL-17F and slL-17RA were reduced in patients with AE. Similarly, the cellular production of lL-17F and slL-L7RA in response to *E. multilocularis* antigens was low in AE patients, while levels of slL-17RB were highly enhanced. These observations suggest immune-modulating properties of *E. multitocularis* on lL-17 cytokine-mediated pro-inflammatory immune responses; this may facilitate the tissue infiltrative growth of the parasite and its persistence in the human host.

## 1. Introduction

Alveolar Echinococcosis (AE) of man can develop following the ingestion of eggs of *Echinococcus multilocularis.* Egg-hatched larvae will migrate into various host tissues, mainly liver, and their proliferative and tissue-infiltrative growth as metacestode larvae will cause damage and ultimately organ failure. Dissemination of cells of metacestode larvae may initiate metastasis-like parasite growth in secondary organs such as lungs and CNS which impairs surgical resection [1]. Since current chemotherapy with imidazoles is only parasitostatic, AE cases with inoperable parasite lesions require life-long medication [2]. 

In some cases of human AE, a spontaneous healing of the disease was observed [1, 3]. Such abortive cases are characterized by calcified parasite lesions suggesting the generation of immune responses which are able to limit parasite growth in humans [4]. Previous studies have shown that Th1- and Th2-type immune responses might be important for clearance of the infection and are associated with the chronic and progressive course of disease [4]; however, knowledge about the crucial determinants which limit parasite growth and disease progression remains scarce. IL-5 is the predominant cytokine expressed by PBMCs in AE patients [5], and Th2-type IL-3, IL-5, and IL-10 were enhanced in severely ill AE patients [6–8] while *E. multilocularis* antigen-induced IFN-*γ* and spontaneous IL-12 production were decreased [9, 10]. Most important, IL-12 and IFN-*γ* inhibited larval growth and metacestode dissemination in *E. multilocularis-*infected mice [11, 12], while the application of IFN-*γ* stopped disease progression in an AE patient [13].

Th1- and Th2-type immune responses in AE have extensively been studied, but pro-inflammatory and regulatory chemokines as well as Th17-type cytokines have received less attention. Immune responses against metacestode larvae of *E. multilocularis* will create persistent sites of inflammation and the formation of peri-parasite granulomas. The chemokines CCL3/MIP-1*α*, CCL4/MIP-1*β*, and CCL5/RANTES were highly elevated in AE patients [14], while mononuclear cells isolated from peri-parasite host granulomas secreted high amount of IL-10 and low amounts of IFN-*γ* disclosing an immune regulation which will counteract inflammatory responses in AE patients [15].

The role of IL-17 cytokines and Th17-type immune responses in AE disease is yet unexplored. The six family members identified (IL17A-F) exert mostly pro-inflammatory activities [16]. IL17A and IL17F, mediators of the recently described proinflammatory Th17-type immune responses, have been associated with inflammatory disorders like rheumatoid arthritis and inflammatory bowel disease [17, 18] but also with protection against extracellular bacteria and fungi [19, 20]. We analyzed levels of pro-inflammatory IL-17 members (IL-17A, IL-17B and IL-17F) as well as their soluble common receptors (IL-17RA and IL-17RB) in clinically staged AE patients, that is, cured, stable, and progressive AE, and in infection-free controls. The altered concentrations of IL-17B, Th17-type cytokine IL-17F, and their soluble receptors at distinct stages of AE disease suggest that these pro-inflammatory cytokines may contribute to the clinical outcome of *E. multilocularis *infection.

## 2. Materials and Methods

### 2.1. Study Groups

The patient cohort consisted of 93 patients (58 females/35 males) diagnosed with Alveolar Echinococcosis at University of Ulm Clinics/Germany. The AE patients' mean age was 57 years, ranging from 17 to 83 years. Blood samples from 12 AE-free individuals from the Blood Transfusion Centre at University Clinics Tübingen served as controls. The UKT Tübingen and University of Ulm Clinics are situated in the federal state of Baden-Württemberg of Germany, a region endemic for *E. multilocularis *infections. In the AE patient groups, 23 cases were diagnosed with cured, 64 with stable, and 6 with progressive AE. The classification of AE patients in different clinical stages of AE was accomplished according to the World Health Organization- (WHO-) PNM (P = parasitic mass in the liver, N = involvement of neighboring organs, and M = metastasis) system previously published by Kern and coworkers [21]. Curative resection, stable disease, progressive disease, or presence of an apparently dead, fully calcified lesion was established by magnetic resonance imaging on the basis of lesion size and morphology at the respective follow-up intervals. This classification has been used for follow-up studies of AE patients [22, 23]. Written consent was obtained from all participating patients, and this study was approved by the Ethics Review Board at University of Ulm Clinics (Ethik-Kommissions Antrag number 71/2004).

### 2.2. *In Vitro* Culture of Echinococcus Multilocularis Metacestodes

*E. multilocularis* metacestodes were cultivated at 37°C, 5% CO_2_ and saturated humidity as previously described [14]. For the generation of single-cell lines, *in vitro* maintained *E. multilocularis *metacestode tissue blocks were cut into small pieces and cultured in RPMI 1640 supplemented with 5% FCS and 1% antibiotic-antimycotic solution (PAA, Cölbe, Germany) in cell tissue culture flasks at 37%, 5% CO_2_, and saturated humidity. After 3 days cell culture flasks were washed with RPMI supplemented with antibiotics (as above) to obtain flask surface-adherent *E. multilocularis-*derived cells. Adherent *E. multilocularis *single-cells (EmZ) were grown as above and flask cultures were split when cell overgrowth was observed. Cells were harvested, centrifuged, and stored at −80°C for further use.

### 2.3. Antigen Preparation

The preparation of *E. multilocularis *metacestode and *Ascaris lumbricoides* antigens was performed as described previously [14]. Briefly, *E. multilocularis* metacestode tissues or adult *A. lumbricoides* were homogenized using a Ten Broek tissue grinder and subsequently ultrasonified (30% intensity, pulse 1 second for 8 minutes). The *Echinococcus* metacestode or *Ascaris* adult worm suspensions were then centrifuged at 4°C, sterile filtered (0.22 *μ*m) and kept at −20°C. For *E. multilocularis* vesicle antigen preparation, entire *E. multilocularis* vesicles were collected separated from *in vitro *culture medium, and vesicles were ruptured by sonication pulses (30% intensity, pulse 1s for 1 min). Such disrupted vesicles were then homogenized, that is, grinded with a Ten-Broek tissue grinder on ice until a homogenous liquid extract was produced, then sonicated again (30% intensity, pulse 1 s for 8 min) and thereafter the vesicle homogenate was centrifuged at 5000 g for 30 min at 4°C. The supernatant was sterile filtrated (0.22 *μ*m) and stored at −70°C. For single-cell *E. multilocularis* antigen preparation, *in vitro* grown adherent *E. multilocularis* single-cells were detached from the culture flask surface, and were collected and separated from *in vitro* culture medium by centrifugation (1.500 g for 5 minutes). The cell pellet was homogenized, that is, grinded with a Ten-Broek tissue grinder on ice until a homogenous liquid extract was produced, then sonicated (30% intensity, pulse 1 s for 8 min) and thereafter the cell homogenate was centrifuged at 5000 g for 30 min at 4°C. The supernatant was sterile filtrated (0.22 *μ*m) and stored at −70°C. Protein concentrations were determined by bicinchoninic acid (BCA) protein determination (Pierce, Rockford, IL, USA). *Entamoeba histolytica* antigen (EhAg) was a kind gift of B. Walderich (Institute for Tropical Medicine, Tübingen, Germany).

### 2.4. Isolation of Peripheral Blood Mononuclear Cells (PBMC)

PBMC and plasma from AE patients and control individuals were isolated by means of Ficoll density gradient centrifugation as described previously [24]. PBMC were adjusted to a concentration of 2 × 10^6^ cells per mL and dispersed into 48 well tissue culture plates with 0.5 mL per well in RPMI 1640 supplemented with 5% FCS and 1% antibiotic-antimycotic solution. PBMC were stimulated for 24 and 48 hours with either 5 *μ*L *E. multilocularis* metacestode antigen (Em, stock concentration 60 *μ*g/mL), *E. multilocularis* single-cell antigen (EmZ, stock concentration 60 *μ*g/mL), *E. multilocularis* vesicle antigen (EmV, stock concentration 60 *μ*g/mL), *Ascaris* antigen (Asc, stock concentration 3.7 *μ*g/mL), or *E. histolytica* antigen (Eh, stock concentration 100 *μ*g/mL) or left unstimulated (base) at 37°C, saturated humidity, and 5% CO_2_. Cells and cell culture supernatant were harvested, separated by centrifugation, and stored at −80°C.

### 2.5. Determination of Cytokine and Chemokine Concentrations

Cell culture supernatants and plasma were stored at −80°C before use. Cytokine and chemokine concentrations were determined by sandwich enzyme-linked immunosorbent assay (ELISA) kits for IL-17A, IL-17B, IL-17F, IL-17RA, and IL-17RB (R&D Systems, MN, USA). The assays were performed according to the manufacturers' guidelines. Conversion of optical densities (OD) to final concentrations (pg/mL) was calculated by using cytokine-specific standard curves. 

### 2.6. Data Analysis and Statistics

The statistical package JMP 9.0 (SAS Institute, Heidelberg, Germany) was used for statistical analyses. Significant differences of cytokine and chemokine concentrations were determined by analysis of variance (ANOVA) and Tukey's test. Due to multiple comparisons the level of significance was adjusted by the Bonferroni-Holm method.

## 3. Results

### 3.1. Plasma Levels of Proinflammatory IL-17 Family Members and Soluble Receptor Components in AE Patients and Infection-Free Controls

Plasma concentrations of pro-inflammatory IL-17 family members IL-17A, IL-17B, IL-17F and their common soluble receptor subunits, sIL-17RA and sIL-17RB, were quantified in AE patients with different states of disease and in infection-free controls. 

**Figure 1:**
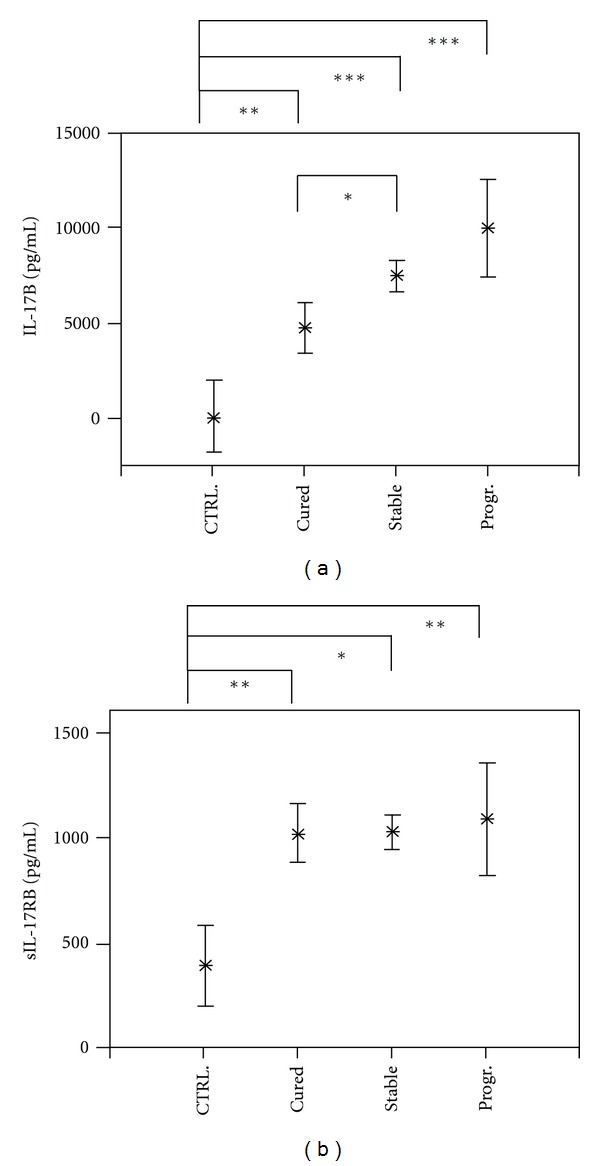
Plasma concentrations of interleukin (IL)-17B (part a) and soluble receptor IL-17RB (part b) in Alveolar Echinococcosis (AE) patients and in infection-free controls (CTRL). The patients were grouped according to their state of infection, that is, cured, stable, or progressive (Prog.) Alveolar Echinococcosis. The plasma concentrations are shown as the mean values in pg/mL with the 95% upper and lower confidence interval. The level of significance was adjusted by the Bonferroni-Holm method. Significant differences between the groups are indicated (*P < 0.05, **P < 0.01, and ***P < 0.001).

**Figure 2:**
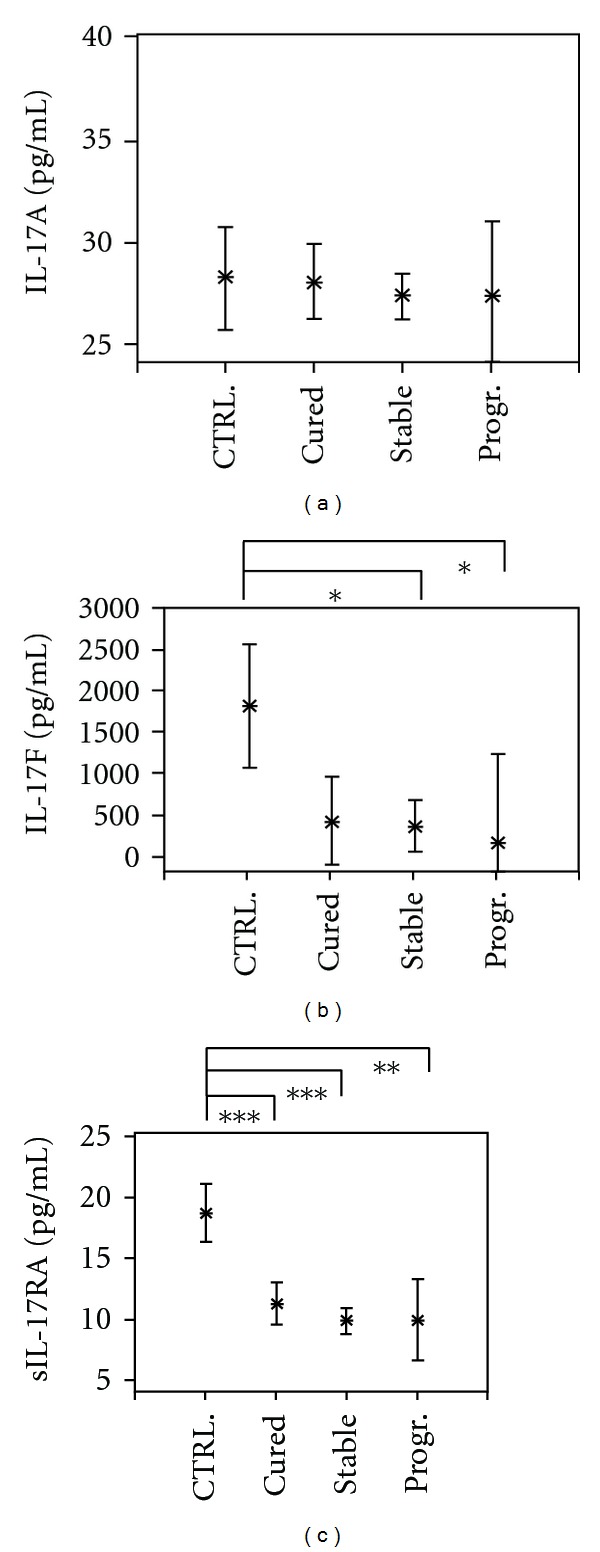
Plasma concentrations of interleukin (IL)-17A (part a) and IL-17F (part b) and of soluble IL-17RA (part c) in Alveolar Echinococcosis (AE) patients and infection-free controls (CTRL). Patients were grouped according to their state of infection, that is, cured, stable, or progressive (Prog.) Alveolar Echinococcosis. The plasma concentrations are shown as the mean values in pg/ml with the 95% upper and lower confidence interval. The level of significance was adjusted by the Bonferroni-Holm method. Significant differences between the groups are indicated (*P < 0.05, **P < 0.01, and ***P < 0.001).

The levels of IL-17B were lowest in healthy controls and were significantly increased in all AE patient groups (*P* < 0.01 and *P* < 0.001) (Figure 1(a)). Within the AE patient group, lowest concentrations of IL-17B were detected in cured cases of AE, while highest concentrations were observed in progressive cases. Soluble IL-17RB levels were lowest in noninfected controls and highly elevated in all AE patient groups, while IL-17RB did not differ between patient groups (Figure 1(b)). Similar plasma concentrations of IL-17A were observed within AE patient groups and infection-free controls (Figure 2(a)). 

In contrast, the concentrations of IL-17F, and its soluble receptor IL-17RA were the highest in infection-free controls. While plasma levels of IL-17F were significantly reduced in stable and progressive cases of AE (*P* < 0.05), significantly decreased levels of soluble IL-17RA concentrations were detected in all AE patient groups (*P* < 0.01 and *P* < 0.001) (Figures 2(b) and 2(c)).

### 3.2. Echinococcus Multilocularis Antigen-(EmAg-) Induced Cellular Production of Soluble IL-17RA from AE Patients and Controls

**Figure 3:**
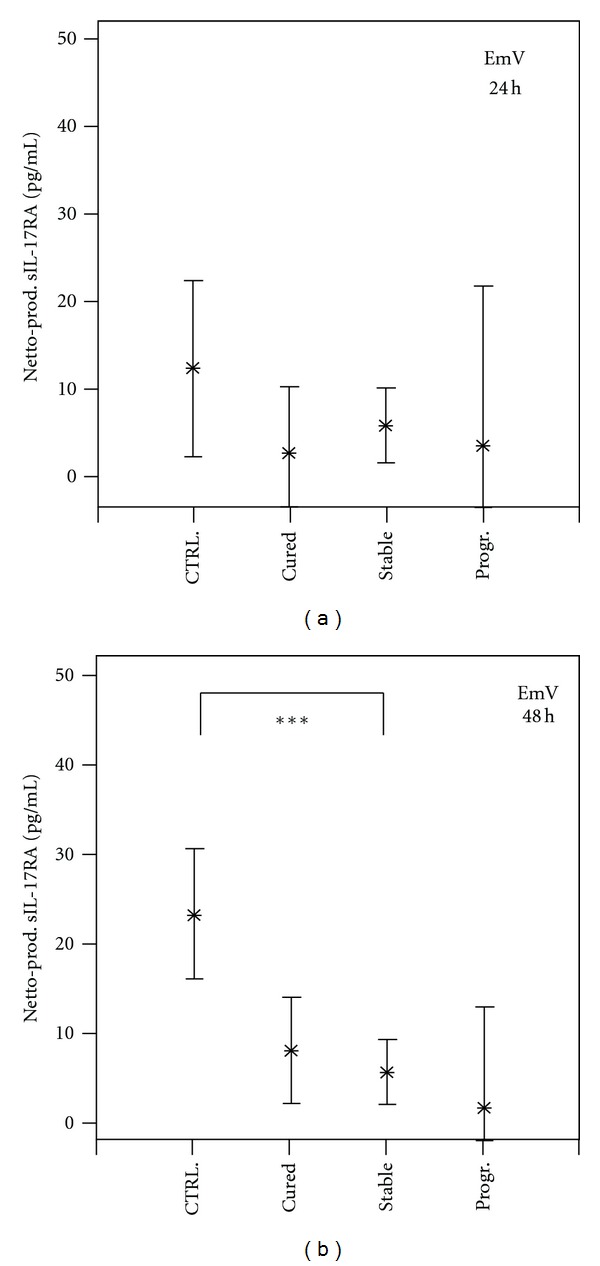
*Echinococcus multilocularis* antigen induced cellular production of soluble interleukin-17 receptor A (sIL-17RA) by peripheral blood mononuclear cells (PBMCs) from Alveolar Echinococcosis (AE) patients and infection-free controls (CTRL). Patients were grouped according to their state of infection, that is, cured, stable, or progressive (Prog.) Alveolar Echinococcosis. PBMCs from patients and controls were stimulated with *E. multilocularis* vesicle extract (EmV) for 24 (part a) and 48 hours (part b) or left without stimulation. Cytokine concentrations in cell culture supernatant were quantified by specific ELISA. The EmAg-induced cytokine production (Netto Prod.) was calculated by subtracting the cytokine production in not stimulated PBMC cultures from EmV-stimulated cytokine production. The cytokine production is shown as mean values in pg/mL with the 95% upper and lower confidence interval. The level of significance was adjusted by the Bonferroni-Holm method. The significant differences between groups are indicated (*P < 0.05, **P < 0.01, ***P < 0.001).

The production of soluble IL-17RA, sIL-17RB, and IL-17F by peripheral blood mononuclear cells (PBMC) was investigated in AE patients and controls. Stimulation of PBMC with *Echinococcus multilocularis* vesicle (EmV) antigen for 24 hours did not result in cellular production differences of soluble IL-17RA between AE patients and infection-free control groups (Figure 3(a)). In addition, no differences within the AE patient groups could be observed. After 48 hours of stimulation a decreased cellular production of sIL-17RA by PBMC from all AE patient groups was observed, with production levels in healthy controls and stable AE cases being significantly different (*P* < 0.01) (Figure 3(b)). Production of cytokines and soluble receptors levels in response to* Ascaris lumbricoides* (AscAg) or to *Entamoeba histolytica* (EhAg) antigens did not differ between AE patient groups and controls.

### 3.3. EmAg-Induced Cellular Production of Soluble IL-17RB from AE Patients and Controls

**Figure 4:**
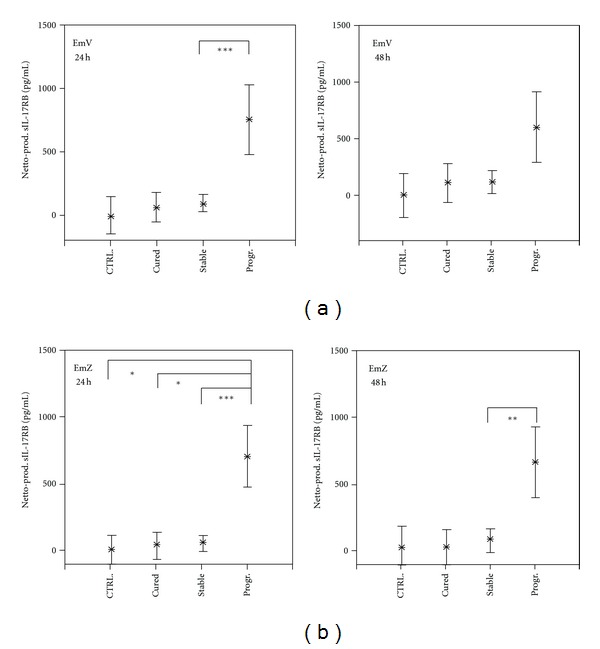
*Echinococcus multilocularis* antigen induced cellular production of soluble interleukin-17 receptor B (sIL-17RB) by PBMC from Alveolar Echinococcosis (AE) patients and infection-free controls (CTRL). Patients were grouped according to their state of infection, that is, cured, stable, or progressive (Prog.) Alveolar Echinococcosis. PBMCs from patients and controls were stimulated with *Echinococcus multilocularis* vesicle extract (EmV) (a) and single-cell extract (EmZ) (b) for 24 and 48 hours or left unstimulated. Cytokine concentrations in cell culture supernatant were determined by specific ELISA. The EmAg-induced cytokine production (Netto Prod.) was calculated by subtracting the cytokine production in not stimulated PBMC (Baseline) cultures from EmV-stimulated cytokine production (Brutto production). The cytokine production is shown as mean values in pg/mL with the 95% upper and lower confidence interval. The level of significance was adjusted by the Bonferroni-Holm method. The significant differences between groups are indicated (*P < 0.05, **P < 0.01, and ***P < 0.001).

PBMC from progressive cases of AE produced high amounts of sIL-17RB following 24 and 48 hour stimulation with *Echinococcus multilocularis *vesicle (EmV) and single-cell (EmZ) extract (Figures 4(a) and 4(b)). The production difference between PBMC from progressive cases and the other groups was more prominent after 24 hours of stimulation than after 48 hours. The cellular production levels of sIL-17RB from infection-free controls and cured and stable cases did not differ in response to EmV or EmZ stimulation. Vesicle components, that is, parts of the laminated and germinal layer, but also hydatid fluid, which constitutes the largest volume of vesicles, may have conferred the observed effects on sIL-17RB production. The single-cell line extract (EmZ) may contain pro-inflammatory components from the inner germinal layer of the metacestode.

### 3.4. EmAg-Induced Cellular Production of IL-17F in AE Patients and Controls

**Figure 5:**
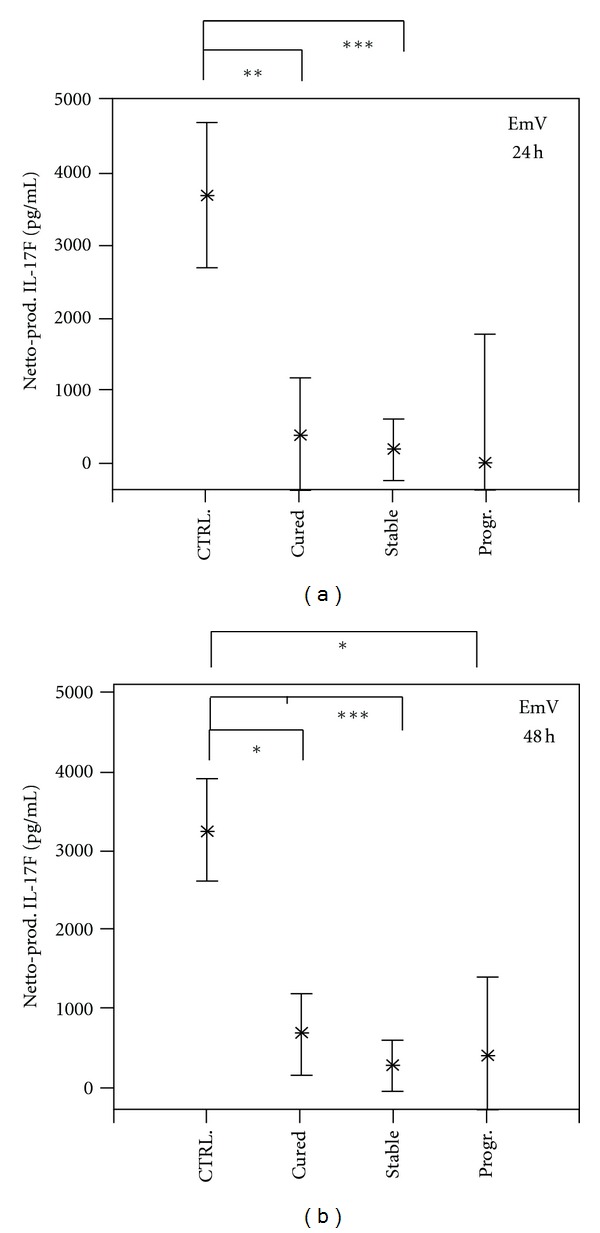
*Echinococcus multilocularis* antigen- (EmAg-) induced cellular production of interleukin-17F (IL-17F) by PBMC from Alveolar Echinococcosis (AE) patients and infection-free controls (CTRL). Patients were grouped according to their state of infection, that is, cured, stable, or progressive (Prog.) Alveolar Echinococcosis. PBMC from patients and controls were stimulated with *E. multilocularis* vesicle extract (EmV) for 24 (part a) and 48 hours (part b) or left unstimulated. Cytokine concentrations in cell culture supernatant were determined by specific ELISA. The EmAg-induced cytokine production (Netto Prod.) was calculated by subtracting the cytokine production in not stimulated PBMC cultures (baseline) from EmV-stimulated cytokine production (Brutto production). The cytokine production is shown as mean values in pg/mL with the 95% upper and lower confidence interval. The level of significance was adjusted by the Bonferroni-Holm method. The significant differences between groups are indicated (*P < 0.05, **P < 0.01, and ***P < 0.001).

Cellular production of IL-17F in response to *Echinococcus multilocularis *vesicle extract (EmV) for 24 and 48 hours was the highest in the control group (Figure 5). Compared to healthy controls, PBMC from all AE patient groups produced significant lower amounts of IL-17F in response to antigen stimulation (*P* < 0.05 and *P* < 0.001) (Figure 5). Stimulation of PBMC with EmV resulted in a similar IL-17F production in the three clinical AE patient groups (Figure 5). The IL-17F production by PBMC in response to *E. multilocularis* single-cell extract showed no difference between the studied groups (data not shown).

## 4. Discussion

Spontaneously healed Alveolar Echinococcosis has been observed [3], and previous works indicate that Th2-type immune responses in AE are associated with progressive AE while proinflammatory Th1-type cytokines are important in protection and disease regression. Therefore, we focused on pro-inflammatory cytokines of the IL-17 family yet uncharacterized in AE and searched whether these immune mediators of inflammation and their receptors associated with progression or regression of AE. In the present study, pro-inflammatory IL-17 cytokine family members and their common receptors disclosed divergent cellular production profiles and plasma levels in AE patient groups and controls; Th17-type IL-17A levels were similar in patients with progressive, stable, and healed *E. multilocularis *metacestode lesions, IL-17B enhanced in AE patients, whilst the Th17-type IL-17F production was highest in controls and depressed in all AE patient groups. Such diverse and opposing cytokine profiles revealed distinct dynamics for each member of the IL-17 family during progression or regression of AE.

While Th17 immune responses may confer protection against infections with bacteria and fungi [25, 26], they may also initiate inflammatory responses which promote immune disorders like inflammatory bowel disease and rheumatoid arthritis [17, 18]. The IL-17A and IL-17F cytokines are best characterized, highly homologous, and were initially allocated similar characteristics. Recent findings, however, disclosed that IL-17F is dispensable for immune disorders like collagen-induced arthritis and experimental autoimmune encephalitis, while effective protection against *Staphylococcus aureus* and *Citrobacter rodentium *infections was dependent on the presence of both IL-17A and IL-17F activity [26]. IL-17F is rather associated with protection while IL-17A seems to contribute equally to both protection and inflammatory disorders [26, 27]. 

In AE patients, irrespective of their stage of infection, IL-17F levels were depressed, and such cellular unresponsiveness and depressed cytokine production to *E. multilocularis *metacestode antigens have previously been observed [5, 6]. Furthermore, depressed IL-17F plasma and production levels persisted irrespective whether AE was cured, stable or progressive, suggesting continuing immune responses against residual parasite products. IL-17A and IL-17F are potent inducers of chemotaxis and inflammation, and both can be induced in PBMC by TGF-*β*, IL-6, and IL-21 secreted from antigen presenting cells (APC) [28, 29]. Both trigger proinflammatory responses by the release of neutrophil activating chemokine CXCL8/IL-8 [16] and of proinflammatory cytokines like TNF-*α*, IL-6 and IL-1*β* [30]. Suppression of Th17 immune responses has been demonstrated in infection models, where *Fasciola hepatica*-infected mice had a decreased production of IL-17 [31] and *Schistosoma mansoni*-infected mice with an elevated IL-17 production presented with a reduced adult worm burden [32]. The IL-17 receptor family consists of five dimer-forming subunits (IL-17RA to IL-17RE). IL-17A and IL-17F share the same receptor subunit IL-17RA [27] and IL-17RA is expressed ubiquitously by all cell types [30]. Plasma concentrations as well as the *E. multilocularis *antigen-induced cellular release of soluble IL-17RA were low in patients irrespective of their stage of AE and this paralleled the low plasma levels and the noninducible cellular release of IL-17A by PBMC from AE patients (data not shown). The observed lessened EmAg-specific IL-17F and sIL-17RA levels in AE patients indicate a parasite-induced unresponsiveness occurring with active *E. multilocularis* infection and such cellular anergy may facilitate survival of the parasite in its host. Parasite antigen-specific cellular anergy was similarly observed in filariasis or schistosomiasis patients, where patent infection, that is, with circulating microfilariae in filariasis or egg excretion in schistosomiasis patient, associated with cellular hyporeactivity to parasite-specific antigens; often the patients' cellular responses were lower than observed in endemic controls [33, 34].

Up to date, little is known about the biological properties of IL-17B. Its expression has been found in the monocyte-derived cell line THP-1, chondrocytes, and neurons [35–37], and IL-17B mRNA was detected in cells of the gastrointestinal tract, including stomach, pancreas, and small intestine [35]. IL-17B signals through binding to a homodimeric IL-17RB complex and induces upon binding the release of pro-inflammatory TNF-*α*, IL-1*β*, and IL-6, CXCL8/IL-8 [36], and the migration of neutrophils to the peritoneal cavity in rats [38]. Protective properties of IL-17B in disease have not yet been reported. In AE patients, plasma concentrations of IL-17B and its soluble receptor IL-17RB were strongly elevated and highest in those with progressive AE. The persistent exposure to growing *E. multilocularis* metacestodes may have triggered the release of IL-17B by cells of the gastrointestinal tract, leading to the recruitment of neutrophil granulocytes into peri-parasite lesions. While the effects of IL-17B are similar to those mediated by TNF-*α*, IL-17A, and IL-1*β*, its potency is limited [39]. The IL-17B-induced infiltration of neutrophils into the peritoneal cavity in rats required much higher concentrations compared to TNF-*α* and it was still considerably less effective than the cell migration induced by IL-17A [38]. The elevated IL-17B production in AE patients disclosed a proinflammatory response triggered by *E. multilocularis *antigens, but potentially not strong enough to limit the progressive parasite growth. The decreasing concentration of IL-17B with an cured AE should be further evaluated as a prognostic marker in AE.

IL-17RB serves as receptor subunit for IL-17B and IL-17E [40], commonly expressed by cells of the intestine, but also by liver, pancreas, lung, and kidneys as well as on Th2 and Th9 cells [27, 30, 40, 41]. Plasma concentrations of soluble IL-17RB were highly elevated in AE with no significant differences between the patient groups, while PBMC from progressive AE cases produced high amounts of soluble IL-17RB in response to *E. multilocularis* antigens. While vesicle extracts will contain large amount of vesicle fluid, the single-cell extract will most likely be derived from the inner germinal layer of the metacestode, and therefore, the heightened proinflammatory IL-17 responses in patients with progressive AE may primarily be induced by vesicle fluid components and germinal cells and engaging the IL-17RB activation pathway. Membrane bound and soluble IL-17RB are inducible in human antigen-presenting cells (APC) upon stimulation with Th2-type cytokines IL-4, IL-10, IL-13 and TGF-*β* [42], and these cytokines are associated with progressive AE [5, 6, 8, 43, 44]. PBMC from AE patients produced elevated levels of Th2 cytokines IL-4, IL-5, and IL-10 upon stimulation with crude *E. multilocularis* antigen [5]. Thus, the cellular production of sIL-17RB in AE patients in response to *E. multilocularis* antigen could result from an EmAg-induced production of Th2 cytokines, which subsequently triggered the release of sIL-17RB. The higher concentrations of sIL-17RB in patients may be a direct consequence of this Th2 polarization associated with chronic AE. The biological functions and importance of soluble IL-17 receptors in AE remain tentative; the soluble IL-17RB could act as decoy receptor for IL-17B. Previous studies have shown that the administration of the soluble IL-4 receptor inhibited IL-4-mediated immune responses [45], while soluble IL-6 receptor amplified the effects of IL-6 [46]. High amounts of circulating IL-17RB, as observed with progressive AE, could counteract the effects of IL-17B thus silencing IL-17B-mediated pro-inflammatory responses in patients. Similar to IL-17F, the high amounts of soluble IL-17RB observed in plasma of patients with cured AE might indicate long-lasting effects of residual or inactive parasite lesions, whilst low levels of circulating soluble IL-17RA together with low IL-17F production may reduce Th17 cytokine-mediated inflammation. 

## 5. Conclusion

In summary, the present work discloses a modulation of proinflammatory IL-17 family members and Th17-type immune responses in AE; the persistently altered production of IL-17 cytokine family members and of their soluble receptors highlights the capacity of the *E. multilocularis* metacestode to exert long-lasting immune modulating effects, and further studies should address the protective and preventive potential of IL-17 cytokines during *E. multilocularis* infection.
